# Long‐Term Acclimation and Membrane Lipid Remodelling Enable Anaerobic Oxidation of Methane at Low‐pH by ‘*Ca*. Methanoperedens vercellensis’

**DOI:** 10.1111/1462-2920.70460

**Published:** 2026-11-09

**Authors:** Vojtěch Tláskal, Reinier A. Egas, Wei Wang, Xiaoxiao Zhao, Martijn Wissink, Maider J. Echeveste Medrano, Kevin W. Becker, Felix J. Elling, Cornelia U. Welte

**Affiliations:** ^1^ Institute of Soil Biology and Biogeochemistry Biology Centre of the Czech Academy of Sciences České Budějovice Czech Republic; ^2^ Department of Microbiology Radboud Institute for Biological and Environmental Sciences, Radboud University Nijmegen the Netherlands; ^3^ National Engineering Research Center for Safe Disposal and Resources Recovery of Sludge, School of Environment Harbin Institute of Technology Harbin China; ^4^ Leibniz‐Laboratory for Radiometric Dating and Isotope Research Christian‐Albrecht University of Kiel Kiel Germany; ^5^ GEOMAR Helmholtz Centre for Ocean Research Kiel Kiel Germany; ^6^ Faculty of Biology/Chemistry & MARUM—Center for Marine Environmental Sciences University of Bremen Bremen Germany

**Keywords:** acidosis acclimation, ANME, *Ca*. Methanoperedens, methanotroph

## Abstract

Anaerobic methanotrophic archaea are key members of the methane cycle, preventing emissions of the potent greenhouse gas into the atmosphere. Previous studies of freshwater anaerobic methanotrophs targeted their activity at circumneutral pH whereas molecular ecology studies detected anaerobic methane oxidizers in acidic environments such as peatlands. However, it remains unclear whether they can adapt to low pH and sustain an effective biological methane filter under acidic conditions. Here, we show that a granular enrichment culture of the freshwater methanotroph ‘*Ca*. M. vercellensis’ remains metabolically active down to pH 5.65 with appropriate acclimation time while it loses activity when experiencing immediate acid stress. Population‐wide acclimation is required to support anaerobic methane oxidation at lower pH. Archaeal lipid analyses revealed an increased abundance of zwitterionic intact polar lipids over anionic lipids. This coincided with structural changes in biomass granules while methane oxidation rate and enrichment proportion of ‘*Ca*. M. vercellensis’ remained stable. These results demonstrate that ‘*Ca*. M. vercellensis’ remains metabolically active at lower pH values, despite increased maintenance energy requirements and the need for cytoplasmic pH homeostasis. Furthermore, they highlight that stress adaptations in slow‐growing microorganisms requires long‐term observation. Our findings provide new insights into methane cycling in acidic ecosystems.

## Graphical abstract

Anaerobic methanotrophic archaea from the genus ‘*Ca*. Methanoperedens’ are slow‐growing microorganisms utilizing a range of electron acceptors such as nitrate. A granular enrichment culture of ‘*Ca*. Methanoperedens vercellensis’ remains metabolically active during stepwise acidification of the medium down to pH 5.65, whereas its activity is inhibited by immediate acid stress. Long‐term acclimation potential of the biomass is enabled by remodelling of membrane lipid composition, a viable strategy for survival in acidic ecosystems.

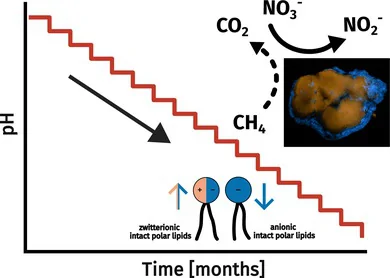

## 1 Introduction

Atmospheric methane (CH_4_) represents an important greenhouse gas, with a 27‐fold stronger warming potential than CO_2_ on a 100‐year timescale (IPCC 2023). Atmospheric methane levels have increased 2.6 times (to 1.91 ppmv in 2022) compared to pre‐industrial values (Lan et al. 2025). While anthropogenic emissions represent a substantial portion of total methane, large quantities are emitted from wetlands and inland freshwater systems (Saunois et al. 2025). Methane is produced by methanogens in anoxic environments with very low redox potentials, such as sediments and gastrointestinal tracts. Anaerobic oxidation of methane (AOM) represents an important biological sink of methane and is performed by anaerobic methane‐oxidizing (ANME) archaea. These microbes are able to use reverse methanogenesis to oxidize methane using a variety of electron acceptors such as sulfate, metal oxides, organic acids and nitrate (N‐DAMO) (Boetius et al. 2000; Raghoebarsing et al. 2006; Haroon et al. 2013; Ettwig et al. 2016; Welte et al. 2016; Cai et al. 2018; Leu et al. 2020; Valenzuela and Cervantes 2021). As an integral part of the global methane cycle, ANME archaea partly offset emissions from natural habitats and act as a biological methane filter. Their activity has been described in marine sediments (Boetius et al. 2000; Wallenius et al. 2025), freshwater sediments (Timmers et al. 2016; Echeveste Medrano, Su, et al. 2025) and wetlands (Valenzuela et al. 2017; Krause and Treude 2021). However, the range of environmental conditions, related to proton stress or electron acceptor availability, at which ANME archaea thrive has not yet been fully explored.

Members of the archaeal family *Methanoperedenaceae* (formerly ANME‐2D, phylum Halobacteriota), with the genus ‘*Candidatus* (*Ca*.) Methanoperedens’ are key methane oxidizers in anoxic freshwater environments (Welte et al. 2016; Liu et al. 2026). Freshwater habitats of ANME, including peatlands, are increasingly impacted by environmental change. While salinization from sea level rise and eutrophication can elevate sulfide concentrations, peatlands are characterized by limited buffering capacity, leading to strongly acidic conditions (pH 3.5–4.5) driven by organic‐matter decomposition and atmospheric nitrogen deposition. Such low pH can markedly affect the physiology and result in cellular adaptations of anaerobic methanotrophs (Gupta et al. 2013; Miller et al. 2019; Barney et al. 2024; Nweze et al. 2024). Mechanisms for coping with low pH involve for example, activity and growth decoupling, increased energy investment to maintenance, membrane remodelling, down‐regulation of protein biosynthesis or increased proton efflux. Furthermore, peat‐soil pH is modulated by seasonal fluctuations, drainage, afforestation‐induced acidification and acid deposition, each imposing additional selective pressures that require methanotrophs to adjust their metabolic traits (Malcolm et al. 2014; Urbanová and Bárta 2016; Kang et al. 2018; Harpenslager et al. 2024).

Studying the physiological response of ANME to environmental changes is challenging due to slow growth (Timmers et al. 2017) and the lack of axenic cultures. Nevertheless, the ability of freshwater anaerobic methanotroph ‘*Ca*. Methanoperedens’ to acclimate to changes in salt concentration and the presence of sulfide has been tested previously (Echeveste Medrano et al. 2024; Echeveste Medrano, Lee, et al. 2025). Accumulation of storage polymers in the cells of ‘*Ca*. Methanoperedens’ contributes to cell stability under osmotic stress (Frank et al. 2023; Echeveste Medrano et al. 2024). Currently, there are, however, limited data available as to how anaerobic methanotrophs react to pH decrease in freshwater, wetland and peatland environments, where widespread AOM has been observed (Smemo and Yavitt 2011; Gupta et al. 2013; Segarra et al. 2015; Barney et al. 2024).

N‐DAMO enrichment bioreactor systems enable physiological studies (McIlroy et al. 2023; Echeveste Medrano et al. 2024; Echeveste Medrano, Lee, et al. 2025; Liu et al. 2026) that have to be interpreted with care due to the presence of a side community consisting of nitrite‐utilizing autotrophs, which detoxify nitrite and heterotrophs thriving off necromass and syntrophic intermediary products (Cai et al. 2019). An advanced enrichment of ‘*Ca*. Methanoperedens’ has been achieved by using electrodes in bioelectrochemical systems as electron acceptors instead of nitrate (Ouboter et al. 2022, 2024). Biofilm enrichment cultures, however, show high sensitivity to a decrease in pH.

Potential adaptation to pH stress in archaea is represented by the ability to modulate the composition of lipids in the archaeal membrane. In environments with high temperature and low pH, archaea modulate membrane fluidity and permeability by synthesizing glycerol dibiphytanyl glycerol tetraether (GDGT) lipids with cyclopentane rings (Zhou et al. 2020). A higher degree of GDGT cyclization (referred to as ring index) leads to tight packing of tetraether lipids which restricts proton influx into the cytoplasm (Chong 2010). A negative correlation of pH and ring index was indeed described in several thermoacidophilic archaeal taxa such as in *Acidilobus* (Boyd et al. 2011), *Picrophilus* (Feyhl‐Buska et al. 2016) or *Sulfolobus* (Cobban et al. 2020) and in hot springs (Boyd et al. 2013). Likewise, the abundance of cyclization genes *grsAB* for GDGT ring synthase A and B (Zeng et al. 2019) was found to be negatively correlated with pH in hot spring metagenomes (Blum et al. 2023). In terms of acid‐adaptation at the level of intact polar lipids (IPLs), Shimada et al. (2008) reported an increase in the number of sugar units in glycophospholipids of the thermoacidophilic archaeon *Thermoplasma acidophilum* . The only study on the influence of pH on intact polar and core lipid adaptation in archaea living at moderate temperatures observed no change in ring index, a small decrease in anionic IPLs and a corresponding increase in IPLs with two sugar units upon decreases in pH within the circumneutral range (Elling et al. 2015). However, membrane lipid adaptation in archaea living in moderate temperature and acidic pH conditions, such as in many wetlands, has not been studied. Core membrane lipids of ‘*Ca*. Methanoperedens carboxydivorans’ (formerly known as BLZ2, Arshad et al. 2015; Berger et al. 2017) and ‘*Ca*. Methanoperedens vercellensis’ are dominated by archaeol and hydroxyarchaeol and contain dihexose as the main component of IPL headgroups (Kurth et al. 2019). The ability of ‘*Ca*. Methanoperedens’ to acclimate to acid stress is, however, unknown.

Based on the environmental diversity of the genus ‘*Ca*. Methanoperedens’, we investigated whether an enrichment culture of ‘*Ca*. M. vercellensis’ can acclimate to a stepwise decrease in pH in a continuous laboratory‐scale bioreactor. The use of ^13^C–CH_4_ as an energy and carbon source (Kurth et al. 2019) allowed assessment of the methane oxidation potential of the enrichment culture under acid stress. Optical microscopy and fluorescent in situ hybridisation (FISH) were employed to examine the morphology of ‘*Ca*. M. vercellensis’ granular biomass. Furthermore, analysis of archaeal lipids and analysis of genes encoding biosynthetic proteins involved in cell membrane composition remodelling were used to investigate the mechanism of ‘*Ca*. Methanoperedens’ acclimation to low pH.

## 2 Methods

### 2.1 Bioreactor Setup and Operation

A 6.5 L bioreactor (Applikon, Delft, The Netherlands) with 4.6 L of liquid phase was anoxically inoculated with 100 mL biomass of a ‘*Ca*. Methanoperedens vercellensis’ enrichment originating from freshwater Italian rice paddy fields (Vaksmaa et al. 2017; Echeveste Medrano et al. 2024; Wissink et al. 2024). The bioreactor was operated as a sequencing fed‐batch reactor (SBR) resulting in granular biomass for 172 days, after which the acidification experiment started and lasted for 246 days. The bioreactor operated on a daily cycle without a settling phase before supernatant removal to stimulate granular biomass formation: each cycle consisted of continuous medium supply (828 ± 93 mL day^−1^) followed by 45 min of supernatant removal. It resulted in a hydraulic retention time of approximately 6.6 days. The medium consisted of 70 mg L^−1^ MgSO_4_·7H_2_O, 100 mg L^−1^ CaCl_2_·2H_2_O and 50 mg L^−1^ KH_2_PO_4_ and was autoclaved prior to the addition of 0.5 mL L^−1^ of trace element solution (Vaksmaa et al. 2017), 0.1 mL L^−1^ of vitamin solution (Vaksmaa et al. 2017) and 16.2 mg L^−1^ of Fe(III) in the form of FeCl_3_·6H_2_O + NTA. The bioreactor contained two standard six‐blade turbines stirring at 200 rpm and was operated at room temperature. Stirring was stopped during supernatant removal to prevent granular biomass washout. The bioreactor was constantly fed with methane with a flow of 10 mL min^−1^ and sparged with Ar:CO_2_ (95/5). The pH was buffered with 1 M KHCO_3_ or HCl solution and controlled by a BL 931700 pH controller Black Stone (Hanna Instruments, Rhode Island, USA). The amount of NaNO_3_ substrate was on average 0.70 ± 0.13 mmol day^−1^ L^−1^ (Figure S1). The experiment was initiated at the standard operating pH of 7.25 (Echeveste Medrano et al. 2024) and was terminated at pH 5.65 with the acidification step of 0.1 set approximately every 7 days (Figure S2).

### 2.2 Bioreactor Batch Activity Assays

To determine the methane oxidation rate of the bioreactor microbial community, the bioreactor was run as a batch with 10% ^13^C–CH_4_ and additional Ar:CO_2_ (95/5) in the headspace to obtain a gauge pressure of 0.18–0.2 bar. The headspace was first flushed for > 1 h with Ar:CO_2_ (95/5) at 40 mL min^−1^ to remove residual methane traces. The starting nitrate concentration was ≈2 mM. Over a period of 5–7 days, labelled ^13^C–CO_2_ and ^12^C–CO_2_ were measured and nitrate limitation, overpressure and a stable pH were controlled. Gas concentrations were measured using 50 μL of bioreactor headspace samples (*n* = 2) at each timepoint by gas chromatography–mass spectrometry (GC–MS) using an Agilent 8890 GC System and Agilent 5977B GC/MSD (Agilent Technologies, USA) equipped with a Porapak Q column (80/100 mesh). The front inlet temperature was 125°C, oven temperature 70°C and TCD temperature 150°C with a run time of 2.5 min. The flow rate of the carrier gas (He) was 10 mL min^−1^. Calibration was performed with standard gas consisting of He/O_2_/N_2_/CH_4_/CO_2_/N_2_O with terminal values of (%): 95.81/1.02/1.03/1.05/1.04/0.050 (Linde Gas Benelux BV, The Netherlands). Ratio of labelled ^13^C–CO_2_ and ^12^C–CO_2_ was used as a proxy for ongoing anaerobic oxidation of methane. Methane oxidation rate was assessed by fitting linear regression to the ^13^C–CO_2_:^12^C–CO_2_ increase as a function of time (*R* ^2^ = 0.88 ± 0.04). Activity assays were normalized by dry weight (*n* = 2, 15 mL). At the end of each batch activity assay, 15 mL of bioreactor biomass were sampled, spun at 3000 rpm for 5 min, supernatant was removed and pelleted biomass was immediately stored at −20°C.

### 2.3 Nucleic Acid Extractions and ddPCR

DNA was extracted from the frozen bioreactor biomass pellets using a phenol‐chloroform extraction method (Angel et al. 2021). For each extraction, 2 mL of bioreactor enrichment culture equivalent were used. The proportion of ‘*Ca*. Methanoperedens’ cells to the rest of the prokaryotic community members in the enrichment culture was determined using droplet digital PCR (ddPCR) on a Bio‐Rad QX200 Droplet Digital PCR System. Quantification of the ‘*Ca*. Methanoperedens’ cells was assessed by targeting the *mcrA* gene with primers mcrA_rev (5′‐CGTTCATBGCGTAGTTVGGRTAGT‐3′, (Steinberg and Regan 2008)) and mlas_mod (5′‐GGYGGTGTMGGDTTCACMCARTA‐3′, (Angel et al. 2011)). The proportion of the side community members was assessed using general 16S rRNA gene primers BAC338F (5′‐ACTCCTACGGGAGGCAG‐3′) and BAC805R (5′‐GACTACCAGGGTATCTAATCC‐3′, (Yu et al. 2005)). ddPCR premix for *mcrA* or 16S rRNA gene quantification contained 11 μL of QX200 ddPCR EvaGreen Supermix, 0.2 μL of each primer (0.1 μM), 8.6 μL of H_2_O and 2 μL of template DNA. PCR conditions of gene amplification were 5 min at 95°C, 5 cycles of 2.5 min at 60°C with 1°C decrease at each cycle, 34 cycles of (30 s at 95°C, 30 s at 55°C and 2 min at 60°C) and 5 min at 4°C followed by 5 min at 90°C (Tian et al. 2026). The non‐specificity of the primers for the 16S rRNA gene was confirmed by in silico annealing to the closed genome of ‘*Ca*. Methanoperedens carboxydivorans' (Egas et al. 2026). The genome contains a single copy of the *mcrA* gene (accession PRJEB98224).

### 2.4 AOM Inhibition in an Activity Assay

To link methane oxidation to ‘*Ca*. Methanoperedens vercellensis’, a batch activity assay with the AOM inhibitor 3‐bromopropanesulfonic acid (3‐BPS) was performed. 3‐BPS is a structural analogue to the 2‐bromoethanesulfonic acid sodium salt (2‐BES). Both are MCR inhibitors and were shown to inhibit the activity of anaerobic oxidation of methane by ‘*Ca*. M. carboxydivorans’ (Wissink et al. 2024). Forty millilitres of aliquots of granular biomass were withdrawn anoxically from the bioreactor (pH = 6.36, experiment day 148) and washed three times in medium through settling followed by liquid removal. The biomass was resuspended in triplicated 120 mL serum flasks in a final medium volume of 40 mL. After closing the serum flasks with red butyl rubber stoppers (Terumo, Leuven, Belgium), the headspace was flushed with N_2_ for 5 min, after which the pressure was set to 1.5–1.6 bar. Medium was supplemented with nitrate to the initial concentration of 1 mM and 3‐BPS was added to half of the bottles to reach a final concentration of 20 mM (Wissink et al. 2024). CO_2_ (0.7% vol/vol final) and ^13^C–CH_4_ (99% stock, 3.3% final vol/vol) were added to the headspace of each flask. pH was adjusted using anoxic HCl and KOH to the final value of 6.49 ± 0.1. Positive controls consisted of medium containing biomass, methane and nitrate. Flasks were incubated at 30°C with shaking at 200 rpm. Following a 1‐h pre‐incubation period, the headspace was analysed regularly over 14 days by GC–MS as described above. The pH was monitored and adjusted as needed throughout the incubation period. Nitrate and nitrite concentrations were measured at regular intervals. To prevent toxic nitrite accumulation, nitrate was replenished to a final concentration of 1 mM based on the consumption rate.

### 2.5 Short‐Term Response Batch Activity Assays

The short‐term tolerance of ‘*Ca*. M. vercellensis’ to acidic conditions was tested in batch bottle activity assays. As inoculum, 40 mL aliquots of granular biomass were anoxically withdrawn from ‘*Ca*. M. vercellensis’ bioreactor (Ouboter et al. 2024). Aliquots were transferred to an anaerobic chamber, washed and resuspended in 40 mL of medium in 120 mL serum flasks as described above. Medium was supplemented with nitrate to an initial concentration of 2 mM. A bacteria‐suppressing antibiotic cocktail containing streptomycin, vancomycin, ampicillin and kanamycin was added to reach a final concentration of 50 μg mL^−1^ each (Wissink et al. 2024). CO_2_ (2% vol/vol final) and ^13^C–CH_4_ (99% stock, 10% final vol/vol) were added to the Ar‐filled headspace of each flask with pressure adjusted to 1.2 bar. To buffer pH, 4‐(2‐hydroxyethyl)‐1‐piperazineethanesulfonic acid (HEPES) buffer at pH 7.25 was used, pH was adjusted using anoxic HCl and KOH to the final value of pH 7.25, pH 6.25 and pH 5.65 (*n* = 3 per pH level). Flasks were incubated at 30°C with shaking at 200 rpm. Following a 2.5‐h pre‐incubation period, the headspace was analysed regularly over 95 h by GC–MS as described above with replicated GC–MS injections (*n* = 2 per bottle per timepoint). Nitrate and nitrite concentrations were measured at regular intervals and nitrate was replenished based on the consumption rate. Methane oxidation rate was assessed by fitting linear regression as described above (*R* ^2^ = 0.85–0.88). Activity assays were normalized by dry weight obtained from 40 mL bioreactor aliquots (*n* = 3).

### 2.6 Intact Polar Lipid Analysis

Lipids were extracted using a modified Bligh & Dyer protocol (Bligh and Dyer 1959; Sturt et al. 2004), dried under a flow of N_2_ and reconstituted in methanol for analysis. Lipids were analysed in a two‐step procedure. First, lipids were identified by ultra‐high performance liquid chromatography (UHPLC) coupled to high‐resolution mass spectrometry (MS). These identifications were used as a reference for lipid identification and quantification of all other samples using a low resolution HPLC‐MS system. The UHPLC–MS system consisted of a Vanquish Horizon UHPLC equipped with a Waters Acquity BEH C_18_ column (2.1 × 150 mm, 1.7 μm particle size) coupled to a Q Exactive Plus high‐resolution, accurate mass Orbitrap MS (Thermo Fisher Scientific, Bremen, Germany) equipped with a heated electrospray ionization source (HESI‐II) operating in positive ionization mode. UHPLC–MS reversed‐phase chromatographic conditions were as described previously (Wörmer et al. 2013). In brief, the injection volume was 10 μL and lipids were eluted at a constant flow rate of 0.4 mL min^−1^ using linear gradients of methanol–water (85:15, v/v; eluent A) to methanol‐isopropanol (50:50, v/v; eluent B), both with 0.04% formic acid and 0.1% NH_3_. The initial condition was 100% A held for 2 min, followed by a gradient to 15% B in 0.1 min and a gradient to 85% B in 18 min. The column was then washed with 100% B for 8 min. The column temperature was 65°C. HESI and MS settings were optimized by infusion of a mixture of lipids into the eluent flow from the LC system using a T‐piece. HESI‐II settings were: capillary temperature, 250°C; sheath gas (N_2_) pressure, 30 arbitrary units (AU); auxiliary gas (N_2_) pressure, 10 AU; spray voltage, 3.5 kV; probe heater temperature, 350°C; S‐lens 75 V. Lipids were detected by scanning from *m/z* 150 to 2000, followed by data‐dependent MS^2^ (isolation window 1.2 *m*/*z*) using resolving powers of 140,000 and 17,500 (FWHM at *m/z* 200) in full‐scan MS and MS^2^, respectively. Optimal fragmentation of lipids was achieved using a stepped normalized collision energy of 15, 25 and 50 eV. A mass calibration was performed using the Thermo Scientific Pierce LTQ Velos ESI Positive Ion Calibration Solution prior to analysis. Lipids were identified by retention time, accurate molecular mass and MS^2^ fragment spectra as described previously (Yoshinaga et al. 2011).

The HPLC‐MS system consisted of an Agilent Series 1260 Infinity high‐performance liquid chromatograph (HPLC) coupled to an Agilent 6130 single quadrupole mass spectrometer (MS). The MS was used in scan mode (200–2000 *m*/*z*) and was equipped with an ESI source operating in positive mode. The HPLC was equipped with an ACE3 C_18_ column (2.1 × 150 mm, 3 μm particle size, Advanced Chromatography Technologies, Aberdeen, Scotland). Chromatographic conditions were as described previously (Zhu et al. 2013). In brief, lipids were eluted at a flow rate of 0.2 mL min^−1^ at 45°C. The HPLC program was as follows: 100% eluent A (methanol: formic acid:14.8 M NH_4_OH, 100:0.04:0.10, v/v/v) for 10 min, followed by a linear gradient to 24% eluent B (2‐propanol:formic acid:14.8 M NH_4_OH, 100:0.04:0.10, v/v/v) in 5 min, followed by a gradient to 65% B in 55 min. The column was then flushed with 90% B for 10 min and re‐equilibrated with 100% A for 10 min. ESI settings were optimized using extracts of *Methanosarcina mazei* Gö1 and *Sulfolobus acidocaldarius* DSM639 and were as follows: drying gas (N_2_) temperature at 350°C, drying gas flow rate at 6 L min^−1^, nebulizer pressure at 35 psi, capillary voltage at 3500 V. Lipids were identified by molecular mass and retention time. Due to a lack of authentic standards, lipid abundances should be considered as semi‐quantitative.

### 2.7 Core Lipid Analysis

Core lipid extracts were prepared by direct acid hydrolysis of biomass (Elling et al. 2014). In brief, cell pellets were dissolved in 3 M HCl in methanol by vortexing (30 s) and ultrasonication (5 min) and then heated for 3 h at 70°C. After cooling to room temperature, ultrapure water was added and the lipids were extracted three times using equal amounts of dichloromethane. Core lipids were analysed using the HPLC‐MS system described above but equipped with an atmospheric pressure chemical ionization interface operating in positive mode. Source conditions were optimized using extracts of *Sulfolobus acidocaldarius* . The source conditions were as follows: drying gas (N_2_) temperature at 200°C, vaporizer temperature at 400°C, drying gas flow rate at 6 L min^−1^, nebulizer pressure at 60 psi, capillary voltage at 3500 V and corona current at 5.0 μA. Lipids were separated at a flow rate of 0.2 mL min^−1^ using two Waters Acquity BEH HILIC columns (2.1 × 150 mm), 1.7 μm particle size in series maintained at 30°C, preceded by a guard column (2.1 × 5 mm) of the same material, as described by (Hopmans et al. 2016). The following linear gradient was used with eluents A (*n*‐hexane) and eluent B (*n*‐hexane:isopropanol, 90:10, v/v): 82% A for 25 min (isocratic), to 35% B in 25 min and finally to 100% B in 30 min. In variation to the method described by Hopmans et al. (2016), the column was flushed using 100% B for 10 min and equilibrated to starting conditions with 18% B for 20 min (Roy et al. 2025).

To detect GDGT ring synthase genes *grsAB* in the genome of ‘*Ca*. M. vercellensis’, amino acid sequences of enzymes GrsA and GrsB from a model organism *Sulfolobus acidocaldarius* (Zeng et al. 2019) were retrieved from Uniprot (Q4J8I0 and Q4JC22 for GrsA and GrsB, respectively). ‘*Ca*. M. vercellensis’ genes were predicted with Prokka v1.14.6 (Seemann 2014) and blastp v2.15.0+ was used to search for candidate *grsAB* genes.

### 2.8 Fluorescence In Situ Hybridization and Optical Microscopy

Biomass (15 mL) was sampled from the bioreactor before the start of the acidification experiment (39 days before, pH = 7.25) and at the end of the experiment (after 246 days, pH = 5.65). Biomass samples were immediately pelleted, supernatant was removed, the cell pellet was resuspended in 500 μL PBS and mixed with 1500 μL of 4% paraformaldehyde. The sample was incubated for 3 h at 4°C and subsequently washed three times with PBS. Washed biomass was resuspended in 1 mL of 1:1 PBS:ethanol (97%) and stored at −20°C until FISH analysis. FISH was performed as described by Wagner et al. (2003) on individual biomass granules from the bioreactor. PFA‐fixed biomass granules were transferred onto a 10‐wells slide and dried for 15 min at 46°C. The slide was dehydrated by dipping in a graded ethanol series for 3 min each (50%, 80%, 100%) and dried at 46°C. Then 10 μL of hybridization buffer was added to the sample followed by 1 μL of each probe. The hybridization buffer contained (per 1 mL): 400 μL formamide, 399 μL Milli‐Q water, 20 μL 1 M Tris–HCl, 180 μL 5 M NaCl and 1 μL 10% SDS. The probe mix contained: Arch915, 5′‐GTGCTCCCCCGCCAATTCCT‐3′ (Stahl 1991) (Fluos; 30 ng μL^−1^) targeting archaea; EUB338‐I, 5′‐GCTGCCTCCCGTAGGAGT‐3′ (Amann et al. 1990) (Cy5; equimolar at 50 ng μL^−1^) targeting bacteria. A nonsense probe NON‐EUB not targeting prokaryotic rRNA (5′‐ACTCCTACGGGAGGCAGC‐3′, Wallner et al. 1993) was applied to samples as a negative control for binding, hybridization carried out without any probe served as a control for autofluorescence. Probe hybridization occurred at 46°C in a humid chamber for 20 h. The slide was then immersed in a wash buffer at 48°C for 15 min, followed by brief immersion in ice‐cold Milli‐Q water and air drying. The wash buffer contained (per 50 mL): 0.46 mL 5 M NaCl, 1 mL 1 M Tris–HCl, 0.5 mL 0.5 M EDTA and 48 mL Milli‐Q water. Dried slides were stored at −20°C until microscopy analysis. Biomass granules were mounted and counterstained with 10 μL of ProLong Diamond Antifade Mountant with DAPI (Thermo Fisher Scientific). Cells were analysed by confocal microscopy with a FV3000 Laser Scanning Microscope (Olympus, Japan) at the Light Microscopy Core Facility, Biology Centre, Czech Academy of Sciences. The micrographs of individual fluorescence channels were merged in Adobe Photoshop.

For optical microscopy of biomass granules, pictures of thawed biomass from the bioreactor with the enrichment culture of ‘*Ca*. M. vercellensis’ were taken on an optical microscope with a millimetre scale bar. Biomass granules of the enrichment culture grown at pH 7.25 and pH 5.65 were analysed.

### 2.9 Statistics

All statistical analyses and visualization were performed using the R statistical language versions 4.3.3 and (R Core Team 2024) and with the packages tidyverse v2.0.0 (Wickham et al. 2019), tidylog v1.1.0 (Elbers 2020), broom v1.0.9 (Robinson and Hayes 2023), see v0.14.1 (Lüdecke et al. 2021), readr v2.1.5 (Wickham et al. 2024), readxl v1.4.5 (Wickham and Bryan 2023), model fitting was performed using base stats and model parameters were displayed using the package report v0.6.1 (Makowski et al. 2023). Mean values ± standard errors are given for experimental groups. Values of *p* ≤ 0.05 were considered statistically significant.

## 3 Results and Discussion

### 3.1 Long‐Term pH Stress in a Laboratory‐Scale Bioreactor Yields a Stable and Active Methane Oxidizing Culture While Short‐Term pH Stress Inhibits Methane Oxidation

To assess whether ‘*Ca*. M. vercellensis’ can tolerate short‐term acid stress without prior acclimation, 5‐day batch activity assays were conducted at three pH levels. While the biomass at pH 7.25 was actively oxidizing methane (0.062 ± 0.009 change in ^45^CO_2_:^44^CO_2_ g_DM_ day^−1^, *n* = 3), methane oxidation at pH 6.25 was almost entirely inhibited (0.003 ± 0.0 change in ^45^CO_2_:^44^CO_2_ g_DM_ day^−1^, *n* = 3). Biomass at pH 5.65 did not show any methane oxidation activity (Figure 1).

**FIGURE 1:**
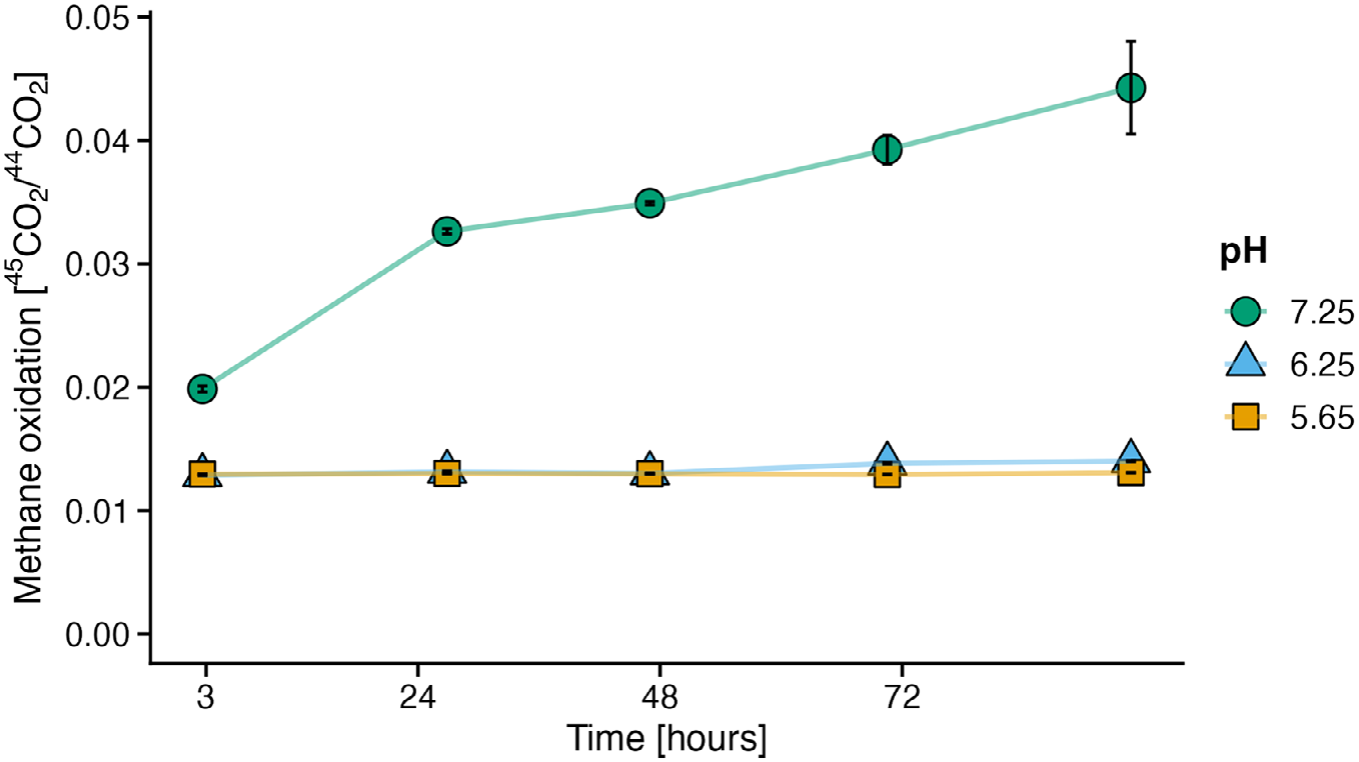
Short‐term response of ‘*Ca*. Methanoperedens vercellensis’ to acid stress. ^45^CO_2_/^44^CO_2_ ratio in the headspace of batch activity assays incubated at pH 7.25, pH 6.25 and pH 5.65 was used to assess the ability to oxidize ^13^C–CH_4_. The headspace composition of activity assays was analysed during 95 h of incubation. Points denote average values ± SE (*n* = 3 biological replicates).

We then assessed whether ‘*Ca*. M. vercellensis’ could acclimate to acid stress during a gradual lowering of the pH during cultivation in a bioreactor (decrease of ~0.1 pH units per week) with continuous medium and substrate supply. During the 225 days of stepwise acidification of the bioreactor medium, the biomass content decreased from 2.33 g L^−1^ to 0.99 g L^−1^ (57.7% decrease, Figure 2A) indicating that a part of the microbial community could not acclimate to the changed pH conditions. Previous metagenome‐based analyses of the parent bioreactor had indicated that ‘*Ca*. M. vercellensis’ was enriched to 54%–75% (Echeveste Medrano et al. 2024; Ouboter et al. 2024) while at the time of this study the enrichment might have been as low as 10% (Wissink et al. 2026). To investigate whether ‘*Ca*. M. vercellensis’ or the accompanying side community were inhibited due to pH stress, we performed quantification of the reverse methanogenesis gene *mcrA* for assessing the abundance of ‘*Ca*. M. vercellensis’ and bacterial 16S rRNA genes for quantifying the side community.

**FIGURE 2:**
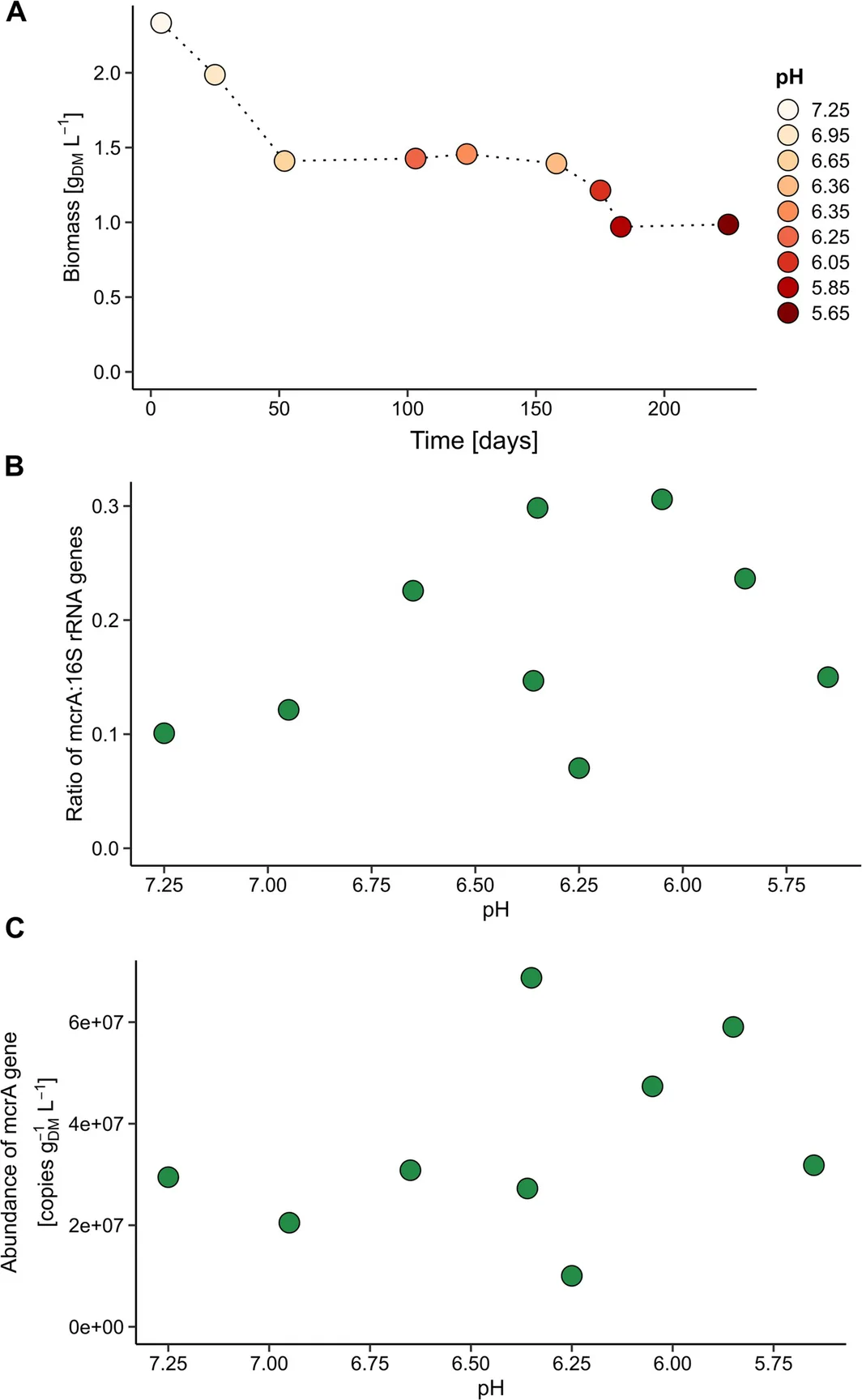
The characteristics of ‘*Ca*. M. vercellensis’ biomass in the bioreactor during the 225 days of the acidification experiment. (A) Decrease of dry weight of total bioreactor biomass per L of bioreactor medium. The colour of points represents pH of the bioreactor medium. (B) Ratio of counts of *mcrA* gene and 16S rRNA gene in DNA samples extracted from the biomass sampled at the end of individual bioreactor batch incubations. (C) Absolute abundance of *mcrA* gene in copies per g_DM_ per L of bioreactor medium as measured in extracted DNA.

The ratio of *mcrA* to the 16S rRNA gene showed mean values of 0.18 ± 0.03 (*n* = 9, Figure 2B), indicating an enrichment of ‘*Ca*. M. vercellensis’ in the microbial community between 10%–30%, while the absolute abundance of the *mcrA* gene showed mean values of 3.61 × 10^7^ ± 6.25 × 10^6^ copies g_DM_^−1^ L^−1^ (*n* = 9, Figure 2C). Both *mcrA*:16S rRNA gene ratio and absolute *mcrA* abundance showed a non‐significant negative correlation with pH (*p* > 0.05). The stable *mcrA* proportion and its absolute abundance together with the observed decline in total bioreactor biomass content point towards a persistence of ‘*Ca*. M. vercellensis’ in the bioreactor, with a possible enrichment towards the end of the bioreactor experiment. Furthermore, the methane oxidation rate (expressed by dry mass corrected change of ^45^CO_2_:^44^CO_2_ ratio in the headspace) was stable throughout the entire experiment and established that slow acclimation to lower pH enabled ‘*Ca*. M. vercellensis’ to remain active until pH 5.65, when the experiment was terminated. The values of methane oxidation rate peaked at pH 5.85 (0.0039 change in ^45^CO_2_:^44^CO_2_ g_DM_ day^−1^) and pH 6.65 (0.0021 change in ^45^CO_2_:^44^CO_2_ g_DM_ day^−1^) while their mean throughout the experiment was 0.0013 ± 0.0004 change in ^45^CO_2_:^44^CO_2_ g_DM_ day^−1^ (*n* = 9, Figure 3A). On day 100, the pH was decreased to 6.25 and associated methane oxidation rate measurements revealed that the methane oxidation activity had dropped compared to earlier measurements, so the pH was increased to pH 6.35 to allow for a prolonged acclimation period before the pH was lowered again (Figure S3). After ~60 days of recovery at pH 6.35 the bioreactor had recovered its methane oxidation activity which permitted further lowering of the pH. The oxidation activity across all samples was in direct relationship to ‘*Ca*. M. vercellensis’ cell numbers as linear regression revealed a significant positive correlation between *mcrA* gene abundance and methane oxidation rate (*p* < 0.05, *R* ^2^ = 0.29, Figure 3B), indicating that ‘*Ca*. M. vercellensis’ was responsible for the observed methane oxidation activity. Cells of ‘*Ca*. M. vercellensis’ contain a single copy of the *mcrA* gene and therefore a proportional link between total cell number and rate of oxidation is expected.

**FIGURE 3:**
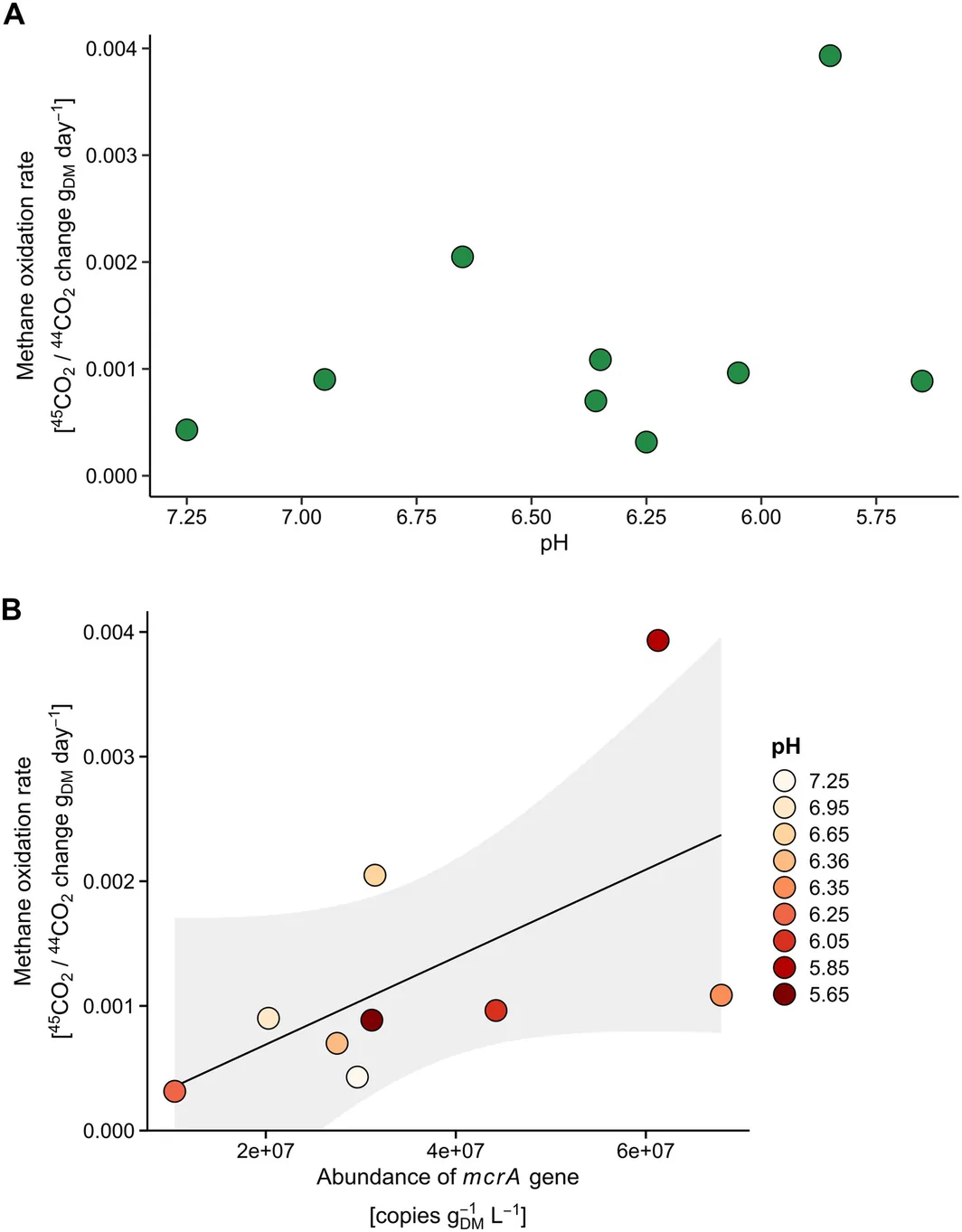
The rate of anaerobic oxidation of methane by the enrichment culture of ‘*Ca*. M. vercellensis’. (A) The rate of anaerobic oxidation of methane is expressed as the slope of change in ratio of ^45^CO_2_/^44^CO_2_ in the bioreactor headspace per day and it is corrected for dry mass of the bioreactor. The measured values of the rate of anaerobic oxidation of methane during the individual bioreactor batch incubations are plotted as a function of pH. (B) Correlation of anaerobic oxidation of methane with the abundance of *mcrA* gene in the bioreactor biomass sampled at individual timepoints. The colour of points represents pH of bioreactor medium, solid lines indicate significant relationships (*p* < 0.05) with 0.95 confidence interval in grey.

To exclude the possibility of interference by other members of the bioreactor microbial community, we performed an inhibition activity assay. Microcosms with the MCR inhibitor 3‐BPS (Wissink et al. 2024) showed inhibition of methane oxidation during the batch activity assay while control microcosms without the addition of 3‐BPS remained active, confirming that ‘*Ca*. M. vercellensis’ was responsible for methane oxidation and that the contribution of bacterial, non‐Mcr methane oxidation was negligible in our experiments (Figure S4). During the long‐term experiment, cells of ‘*Ca*. M. vercellensis’ were under increasing proton stress as the concentration of H^+^ increased 39.8× between the starting point pH 7.25 and the end point pH 5.65. In addition to proton stress, influx of dissolved CO_2_ into the cytoplasm at low pH represents another factor which affects already stressed cells. While at pH 7.25, 89% of CO_2_ is present as bicarbonate (HCO_3_^−^) and uncharged CO_2_ is represented by 11.2%, at pH 5.65 the concentration of CO_2_ increases 7.4× (83.4%). Passive diffusion of this pool of uncharged CO_2_ across cell membranes and subsequent rehydration to HCO_3_^−^ + H^+^ releases protons directly to the cytoplasm, dissipating the proton motive force. As the cytoplasm has a limited buffering capacity, CO_2_ influx represents an additional cellular energy demand. Cells already facing large H^+^ pressure from the extracellular environment need additional proton pumps and chaperons to maintain cytoplasmic pH homeostasis. Despite that, our data collectively indicate that ‘*Ca*. M. vercellensis’ can acclimate to long‐term acidification but is reversibly inactivated by immediate acid stress.

### 3.2 Granular Biomass of ‘*Ca*. M. vercellensis’ Changes in Size and Appearance Upon Acclimation to Low pH

Optical microscopy of ‘*Ca*. M. vercellensis’ biomass granules grown at pH 7.25 and pH 5.65 revealed distinct morphological differences. Biomass granules from pH 7.25 appeared dark with black and red coloration. The red colour might be attributed to the presence of ferritin‐like proteins which were observed in ‘*Ca*. M. carboxydivorans’ (Wissink et al. 2026) and the presence of *c*‐type cytochromes (Arshad et al. 2015; Kletzin et al. 2015). In contrast, biomass granules from pH 5.65 displayed white material on their surface (Figure 4). We speculate that the white material consists of extracellular polymeric substances (EPS) with a composition differing from that observed at pH 7.25 and further analysis is required to characterize it fully. Additionally, acidification was associated with a reduction in biomass granule size, consistent with the overall decline in bioreactor biomass observed over the course of the experiment (Figures 2A and 4). Similar to a previous study, fluorescence in situ hybridization (FISH) visualization revealed that biomass granules consisted of ‘*Ca*. M. vercellensis’ cells accompanied by a bacterial community (Echeveste Medrano et al. 2024). Within the granules, ‘*Ca*. M. vercellensis’ formed densely packed clusters surrounded by bacterial biomass. In biomass granules grown at pH 5.65, the ‘*Ca*. M. vercellensis’ biomass was not fully encapsulated, as portions of some clusters extended to the granule surface, becoming exposed at the outer layer (Figure 4).

**FIGURE 4:**
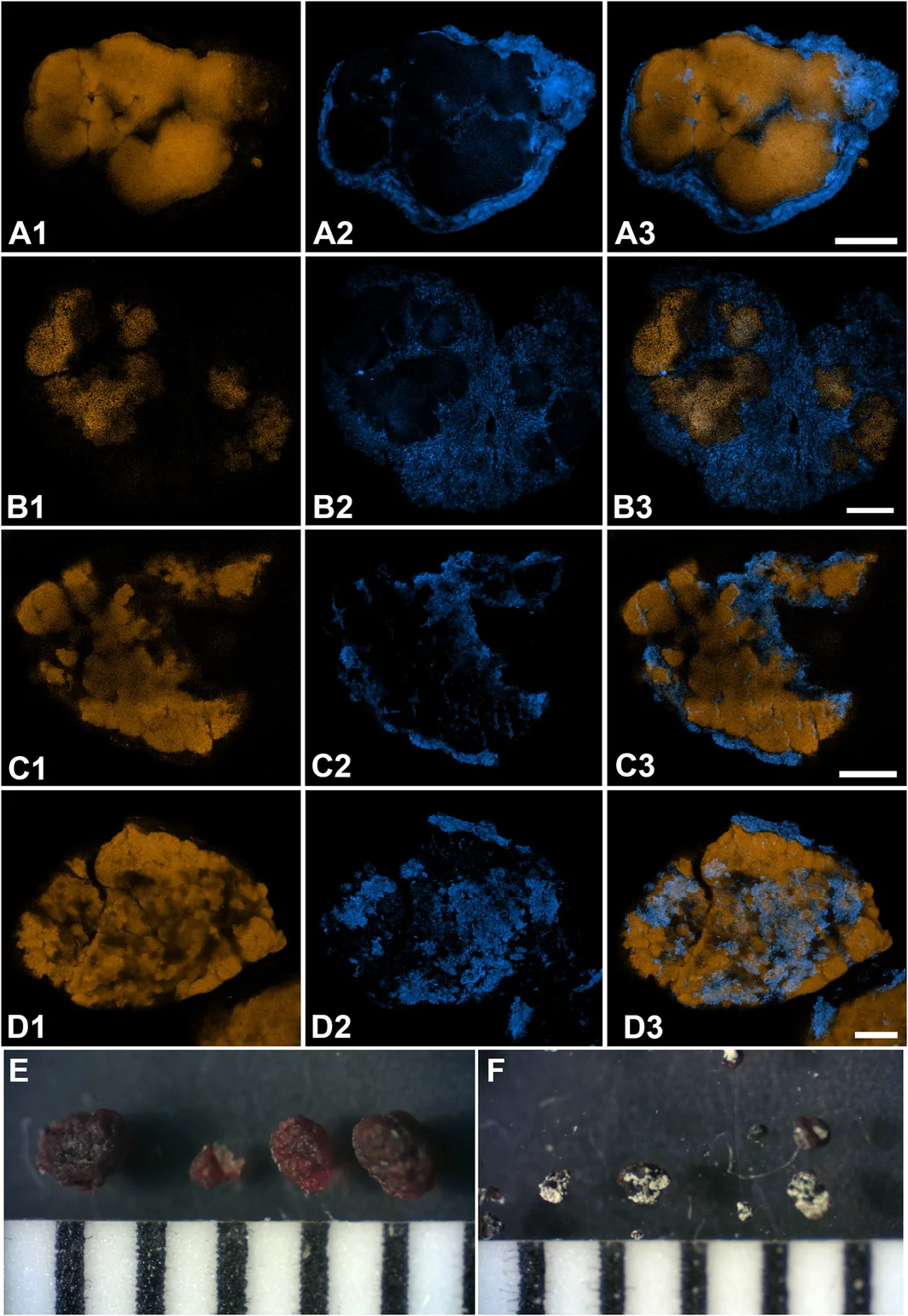
Structure, size and composition of microbial granules from the enrichment culture of ‘*Ca*. M. vercellensis’. Composition of biomass granules is shown in the fluorescent micrographs of samples from the enrichment culture of ‘*Ca*. M. vercellensis’ grown at pH 7.25 (A, B—one layer of a biomass granule) and pH 5.65 (C—one layer of a biomass granule, D—composite layers). In all fluorescent micrographs, orange fluorescence labels ‘*Ca*. M. vercellensis’ (FLUOS‐Arc915), whereas blue fluorescence labels total bacteria (Cy5‐EUBMIX) and the scale bar corresponds to 50 μm. Optical microscope picture of biomass granules of ‘*Ca*. M. vercellensis’ grown at pH 7.25 (E) and pH 5.65 (F). The scale bar in the optical microscope picture has 1 mm steps.

### 3.3 *‘Ca*. M. vercellensis’ Remodels Its Lipid Composition During Acclimation to Low pH

To evaluate the potential of ‘*Ca*. M. vercellensis’ to acclimate to changes in pH, we analysed its genomic capacity to modify its membrane lipids as well as the chemical identity of membrane lipids of biomass originating from the bioreactor. As ‘*Ca*. M. vercellensis’ was the only archaeon in our bioreactor microbial community (Echeveste Medrano et al. 2024; Ouboter et al. 2024; Wissink et al. 2026) we assigned all archaeal membrane lipids to ‘*Ca*. M. vercellensis’. Many archaeal taxa have been found to protect cells from increased proton concentration in the extracellular space by producing monolayer‐forming lipids (GDGTs) and by introducing cyclopentane rings into GDGTs (e.g., Boyd et al. 2011; Cobban et al. 2020). However, blastp search of the genes encoding GDGT ring synthases (*grsAB*; Zeng et al. 2019) revealed its absence from the genome of ‘*Ca*. M. vercellensis’.

We further applied two analytical methods for identification of membrane lipids: (a) Direct acid hydrolysis (Elling et al. 2014), which allows sensitive detection of core lipids including those with cyclopentane rings but partially degrades hydroxyarchaeols and (b) Bligh & Dyer extraction (Bligh and Dyer 1959; Sturt et al. 2004) for milder extraction and subsequent detection of IPLs. The inability for GDGT cyclization by ‘*Ca*. M. vercellensis’ based on gene identification was functionally confirmed by lipidomic analysis of core lipids, which did not detect any GDGTs with cyclopentane rings (e.g., GDGT‐1, GDGT‐2) using either of the extraction and analysis methods. Overall, GDGTs were of low abundance in this dataset and the major membrane lipids of ‘*Ca*. M. vercellensis’ in core lipid and IPL pools were archaeols and hydroxyarchaeols, which are bilayer forming lipids. In further lipidomics analyses, we detected six core lipids (example structures are given in Figure 5A–C) with the most abundant being archaeol (97.27% ± 0.21%), followed by 2OH‐GDGT‐0 (1.00% ± 0.10%) and OH‐GDGT‐0 (0.62% ± 0.08%, Figure 6). Other core lipids showed a mean proportion of total lipid extract < 0.5%. These three most abundant lipids also showed significant relationships with pH (Figure S5). The archaeol proportion was positively correlated with pH (*β* = 0.95, *p* < 0.05), while 2OH‐GDGT‐0 and OH‐GDGT‐0 were negatively correlated (*β* = −0.44 and −0.36, respectively, *p* < 0.05). The observed patterns suggest a decline in archaeol content (by 1.83% overall) accompanied by an increase in hydroxylated core lipids (0.97% and 0.66% for 2OH‐GDGT‐0 and OH‐GDGT‐0, respectively) under lower pH. However, this response was quantitatively limited due to the low relative abundance of these lipids and their importance as adaptations to acid conditions may be small.

**FIGURE 5:**
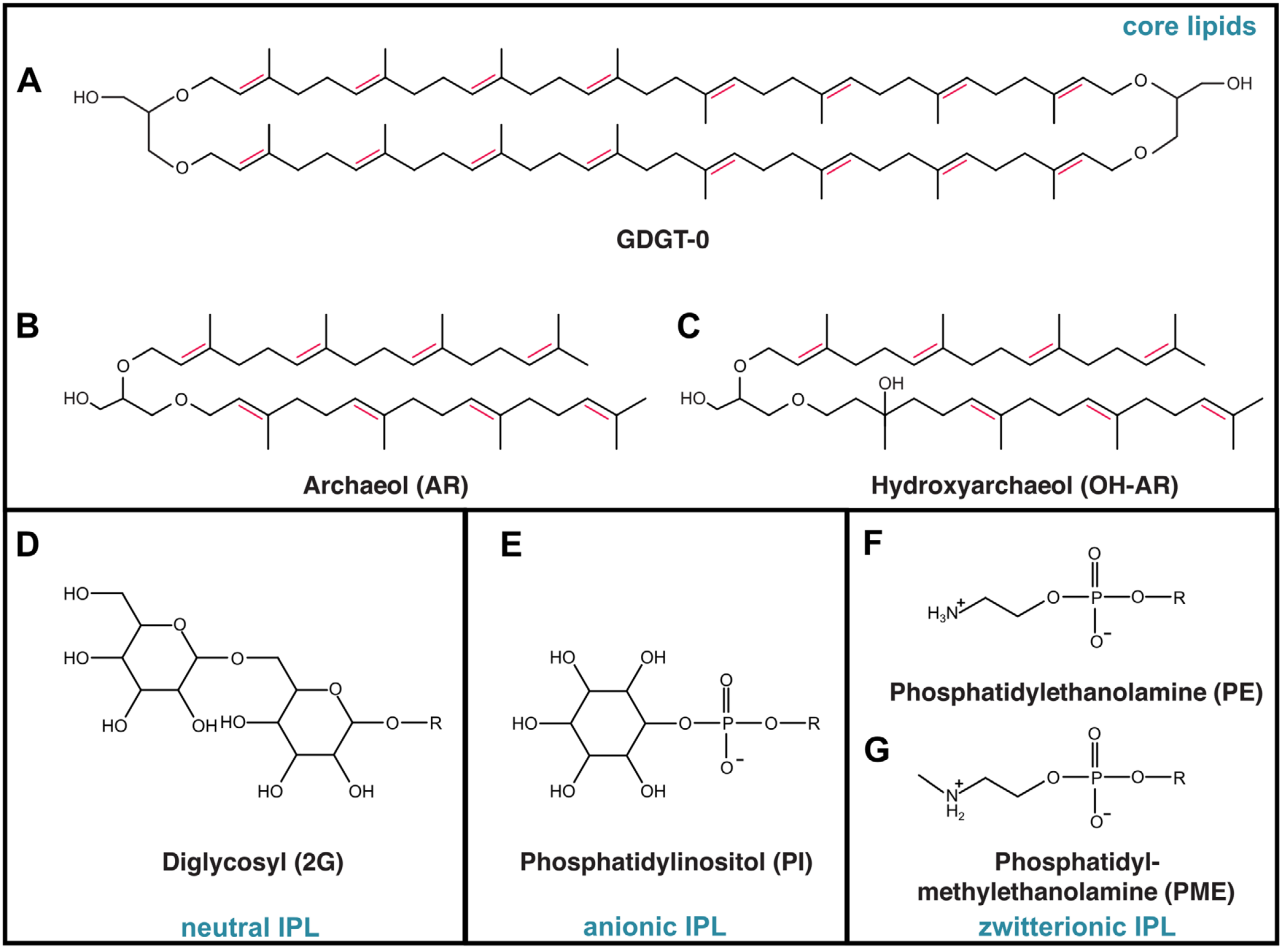
Structures of lipids identified in the biomass of ‘*Ca*. M. vercellensis’. The molecular structures of core lipids (A, B, C), neutral IPL (D), anionic IPL (E) and zwitterionic IPL (F, G) are given. Tentative positions of double bonds in core lipids are given, although the exact positions could not be resolved. Note that not all combinations of IPL headgroup, core lipid and number of core lipid unsaturations occur in ‘*Ca*. M. vercellensis’.

**FIGURE 6:**
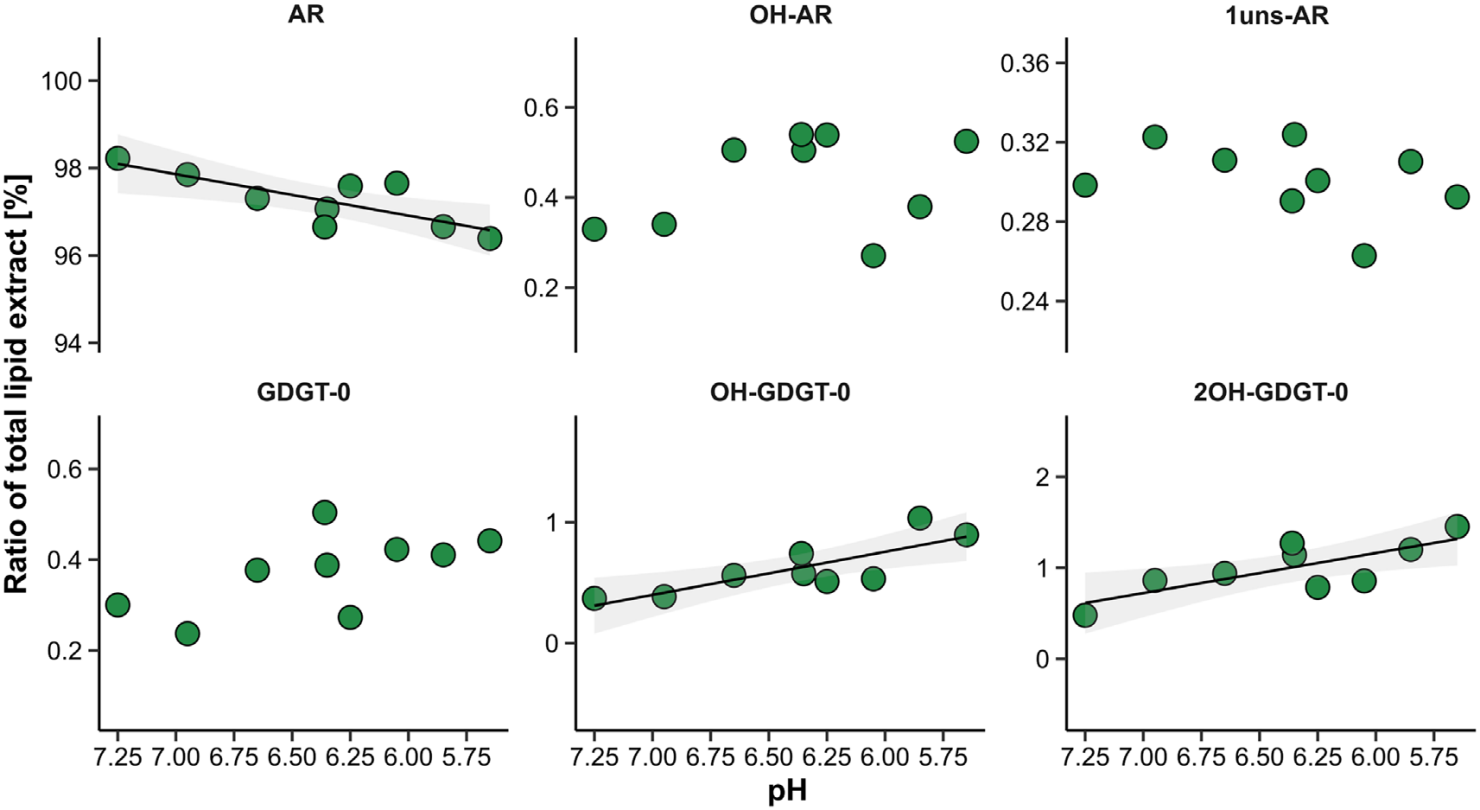
The relative fraction of core lipids extracted from the biomass of ‘*Ca*. M. vercellensis’. Biomass was sampled at the end of each bioreactor batch incubation. The control sample represents biomass before the start of acidification experiment. Core lipids were extracted by the direct acid hydrolysis. Solid lines indicate significant relationships (*p* < 0.05) with 0.95 confidence interval in grey. AR, archaeol.

In contrast to many studies on monolayer‐forming lipids, how archaea with predominantly bilayer‐forming lipids adapt to low pH has not been studied previously. Analysis of IPLs of ‘*Ca*. M. vercellensis’ using Bligh & Dyer extraction protocol showed a range of archaeols and hydroxyarchaeols with different headgroups, which were grouped based on the charge of their headgroups: neutral, anionic and zwitterionic. Examples of identified IPLs are denoted in Figure 5D–G, with MS fragmentation spectra of newly described IPLs shown in Figure S6 and their full list is given in Table S1. The summed relative abundance of IPLs at the level of these three groups showed that the majority of IPLs are zwitterionic or anionic lipids (44.88% ± 2.81% and 31.13% ± 1.84%, respectively). The change in the fraction of total IPLs for these two groups showed a significant trend with acidification of the bioreactor medium (Figure 7). While anionic IPLs (phosphatidylinositol‐archaeol and phosphatidylinositol‐hydroxyarchaeol) were positively correlated with pH (*β* = 8.95, *p* < 0.01), zwitterionic IPLs showed strong negative correlation with pH (*β* = −15.60, *p* < 0.001). Two IPLs were responsible for the observed increase of zwitterionic IPLs with acidification: phosphatidylethanolamine‐1uns‐hydroxyarchaeol and phosphatidylethanolamine‐archaeol (*β* = −12.13 and −1.77, respectively, *p* < 0.001, Figure S5, Table S2). The fraction of neutral IPLs (mainly diglycosyl‐archaeol and diglycosyl‐hydroxyarchaeol) showed a slight increase with pH (*β* = 2.75, *p* < 0.01, 10.29% ± 0.56%).

**FIGURE 7:**
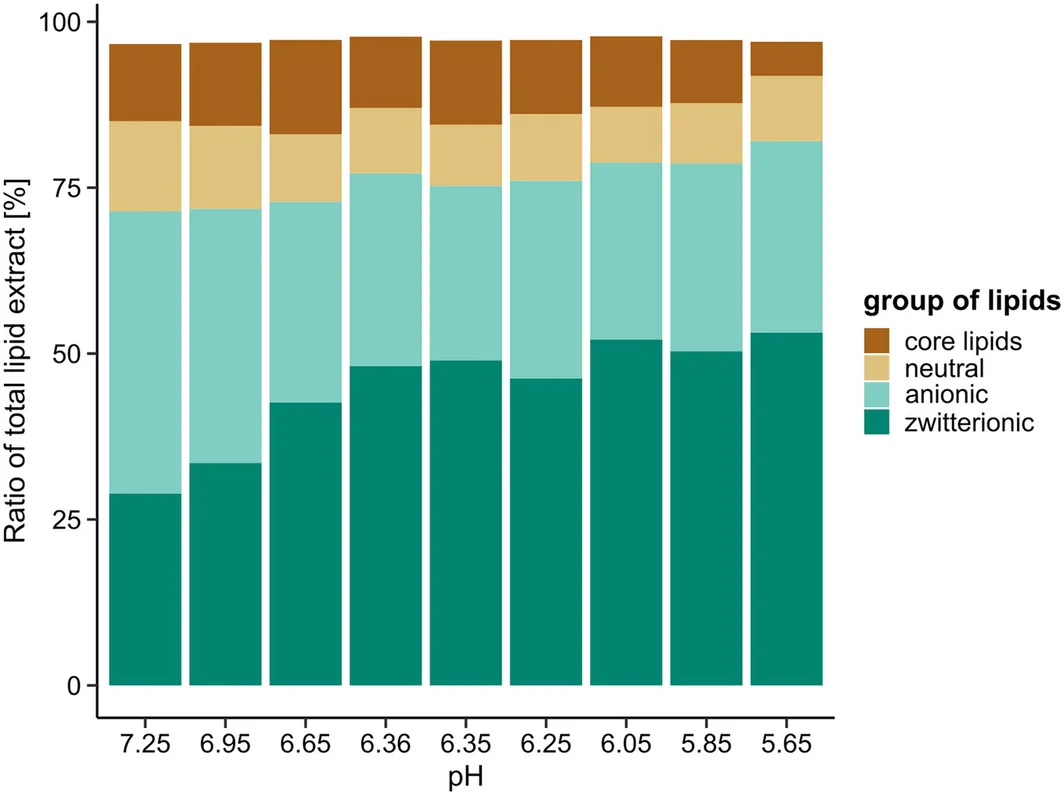
The relative fraction of intact polar lipids extracted from the biomass of ‘*Ca*. M. vercellensis’. Detected lipids are grouped based on their properties given in Table S1. Quinones MK8:8 and MK8:7 identified in the data were omitted from the barplot. The biomass samples were obtained at the end of the respective bioreactor batch incubations at the pH displayed on the *X*‐axis.

Based on the results, we suggest that membrane lipid remodelling through an increased proportion of zwitterionic IPLs represents an acclimation mechanism by ‘*Ca*. M. vercellensis’ in response to low pH. IPL acclimation of archaea to changes in pH has only been studied in a few species, such as *T. acidophilum* (Shimada et al. 2008) and *N. maritimus* (Elling et al. 2015). In both studies higher relative abundances of multiple sugar‐containing IPLs were observed at lower pH values, while the charge state of lipids has not been investigated and discussed. However, analogies can be drawn to the similar IPL headgroups found in bacteria and their acclimation mechanisms. In bacteria, IPL headgroup composition and thereby membrane lipid charge, is modulated to retain membrane fluidity, proton permeability and hydration among other factors (Zhang and Rock 2008). A decrease in extracellular pH, that is, an increase in extracellular H^+^/H_3_O^+^ concentration, may impact surface properties of lipid membranes and reduce the efficiency of proton translocation across the membrane. Although both zwitterionic and anionic IPLs are needed for proper membrane functioning in bacteria (Zhang and Rock 2008), an increase in zwitterionic IPLs may be beneficial under low pH conditions. The small headgroup size and conical shape of phosphatidylethanolamine (PE) IPLs leads to a tight packing of the membrane (Murzyn et al. 2005), which may decrease H^+^ permeability. By contrast, the anionic, phosphatidylinositol (PI) IPLs of ‘*Ca*. M. vercellensis’ are bulky and thus result in a looser packing of the membrane (Peng et al. 2012). Although both PE‐ and PI‐containing IPLs can form hydrogen bonds with each other (Van Den Brink‐van Der Laan et al. 2004; Graber et al. 2018), membranes with increased amounts of PE‐containing IPLs may still retain high levels of hydrogen bonding through the PE‐PE bonding (Hauser et al. 1981; Boggs 1987), thereby decreasing H^+^ permeability. Still, some amount of PI‐containing IPLs or other IPLs are required as the conical shape of PE induces curvature stress (Van Den Brink‐van Der Laan et al. 2004) and membranes solely composed of PE‐containing IPLs would not form stable bilayers (Cullis and De Kruijff 1979). Finally, zwitterionic IPLs are less sensitive to H_3_O^+^ binding and dehydration compared to anionic IPLs, preserving membrane hydration at low pH (Abhinav et al. 2022).

We therefore suggest that ‘*Ca*. M. vercellensis’ acclimates to low pH by controlling proton permeability and hydration of its membrane through changes in lipid headgroup composition, similar to adaptive mechanisms known from bacteria. It remains unconstrained how this acclimation mechanism is regulated in ‘*Ca*. M. vercellensis’. In bacteria, biosynthesis of anionic vs. zwitterionic IPLs may be regulated at the phosphatidic acid branching point (Zhang and Rock 2008). In archaea, the biosynthesis of IPL headgroups is largely unconstrained but it is thought that cytidine diphosphate‐archaeol is a central branching point (Morii and Koga 2003; Morii et al. 2009) and therefore the upregulation of phosphatidylethanolamine‐archaeol biosynthesis could directly impact the biosynthesis of the phosphatidylinositol‐based anionic IPLs of ‘*Ca*. M. vercellensis’.

## 4 Biotechnological and Environmental Implications

Recent studies have been focusing on the occurrence and application of ‘*Ca*. Methanoperedens’ in engineered systems like wastewater treatment plants or landfills. In this study, we show a wider pH activity range which might allow usage of ‘*Ca*. Methanoperedens’ as a bioactive agent in CH_4_‐contaminated wastewater streams at lower pH values (Liu et al. 2020) or landfill cover soils (Li et al. 2024). For example, anaerobic oxidation of methane in laboratory scale bioreactors has been shown to achieve high nitrogen removal from synthetic wastewater (Fan et al. 2020). Similar laboratory setups often operate at stable pH (Liu et al. 2020, 2023); the microbial community in real‐world applications is, however, subjected to dynamic water influent. Activity at low pH would also allow for survival and CH_4_ emission offsetting in wetland and peatland habitats containing nitrate, nitrite, iron or manganese (Segarra et al. 2015). The latter habitat additionally contains high contents of humic compounds which have been shown to support AOM as regenerable electron acceptors (Klüpfel et al. 2014; Valenzuela et al. 2017; Bai et al. 2019). However, AOM in acidic environments remains understudied (Gupta et al. 2013) and further research is needed to quantify its contribution to CH_4_ turnover in these habitats.

When considering acidic natural habitats of ‘*Ca*. M. vercellensis’, its cytoplasm is facing increased proton stress either due to low pH conditions outside the cells or due to CO_2_ rehydration in the cytoplasm. The cytoplasm can further accumulate protons and anions as a result of diffusion and subsequent dissociation of permeable fermentation end products from the surrounding anoxic environment (Russell 1992). Increased concentrations of sulfide in salinized freshwater systems represents another environmental stressor for ‘*Ca*. M. vercellensis’ which might cause separation of the activity and growth at the cellular level as demonstrated for sulfate reducers (O'Flaherty et al. 1998). And as previously shown, growth rate can be linked to lipid membrane remodelling under fluctuating pH (Cobban et al. 2020). Existing studies have reported an increasing GDGT ring index with decreasing pH in archaeal cultures and at the habitat level (Boyd et al. 2011, 2013; Cobban et al. 2020). Our results show that despite the absence of the known gene repertoire for GDGT cyclization (Zeng et al. 2019) ‘*Ca*. M. vercellensis’ maintains unchanged AOM rates under long‐term acid stress and employs changes in IPL composition to acclimate to the stress. Thus, similarly as in the case of bacteria (Zhang and Rock 2008), lipid headgroup composition of ‘*Ca*. M. vercellensis’ in combination with longer acclimation can support continued activity and possibly growth, under pH conditions where growth is otherwise constrained.

‘*Ca*. M. vercellensis’ has previously been shown to acclimate to osmotic stress through the expression of salt‐stress genes leading to osmolyte‐mediated adjustment of cytoplasmic osmotic pressure, accompanied by utilization of the storage polymer polyhydroxyalkanoate (Echeveste Medrano et al. 2024). Another acclimation of freshwater ‘*Ca*. Methanoperedens’ is represented by tolerance against long‐term sulfide exposure (Echeveste Medrano, Lee, et al. 2025). Here, we describe its potential to acclimate to low pH via remodelling of membrane lipid composition and structure of granular biomass. Together, these mechanisms allow methane oxidation under low pH conditions, thereby expanding the environmental niche within which ‘*Ca*. Methanoperedens’ can influence CH_4_ fluxes.

## Author Contributions

**Vojtěch Tláskal:** conceptualization, investigation, funding acquisition, writing – original draft, methodology, visualization, formal analysis. **Reinier A. Egas:** investigation, writing – review and editing, methodology, resources. **Wei Wang:** investigation, writing – review and editing. **Xiaoxiao Zhao:** investigation, visualization, resources, methodology. **Martijn Wissink:** investigation, validation, resources, writing – review and editing. **Maider J. Echeveste Medrano:** investigation, validation, resources. **Kevin W. Becker:** investigation, validation, resources, formal analysis, writing – review and editing. **Felix J. Elling:** conceptualization, investigation, funding acquisition, validation, resources, writing – review and editing. **Cornelia U. Welte:** conceptualization, funding acquisition, writing – review and editing, project administration, supervision, resources.

## Funding

This work was supported by the SIAM Gravitation Grant (024.002.002) and a VIDI Talent Grant (VI.Vidi.223.012) by the Dutch Science Foundation to C.U.W. It was furthermore supported by an ERC Consolidator grant (electroANME, grant number 101229409) to C.U.W. V.T. was supported by the Czech Science Foundation grant number 23‐07434O. F.J.E. and X.Z. were supported by Deutsche Forschungsgemeinschaft grants 441217575 and 527682349. W.W. was supported by the National Natural Science Foundation of China (grant number 52400025) and Science Foundation of National Engineering Research Center for Safe Disposal and Resources Recovery of Sludge (grant number Z2024B009). The authors used services of the Czech‐BioImaging research infrastructure, specifically the Light Microscopy Core Facility, Biology Centre CAS, supported by project LM2023050 funded by the Ministry of Education, Youth and Sports of the Czech Republic, with the instrumental equipment co‐financed by the European Union.

## Conflicts of Interest

The authors declare no conflicts of interest.

## Supporting information


**Figure S1:** The characteristics of the bioreactor with the enrichment culture of ‘*Ca*. M. vercellensis’ during the 225 days of the acidification experiment. Nitrate added to the bioreactor (in mmol day^−1^, A) and concentrations of nitrite (B) and nitrate (C) measured in situ in the bioreactor over the course of the acidification experiment (both in mmol L^−1^).
**Figure S2:** The decrease of pH of bioreactor medium with ‘*Ca*. M. vercellensis’ enrichment culture. Individual points represent timepoints at the beginning of bioreactor batch incubations. The numbers denote pH values.
**Figure S3:** The rate of anaerobic oxidation of methane by the enrichment culture of ‘*Ca*. M. vercellensis’ as a function of time. The rate of anaerobic oxidation of methane is expressed as the slope of change in ratio of ^45^CO_2_/^44^CO_2_ in the bioreactor headspace per day and it is corrected for dry mass of the bioreactor. Individual bioreactor batch activity assays were performed during the 225 days of the acidification experiment.
**Figure S4:** The inhibition of ‘*Ca*. M. vercellensis’ by 3‐bromopropanesulfonic acid (3‐BPS). ^45^CO_2_/^44^CO_2_ ratio in the headspace of batch incubations with 3‐BPS (*n* = 3 biological replicates) and control bottles (*n* = 3 biological replicates) was used to assess oxidation of ^13^C–CH_4_. Replicates are displayed separately to show the variation of individual batch incubation assays.
**Figure S5:** The relative fraction of intact polar lipids extracted from the biomass of ‘*Ca*. M. vercellensis’. Intact polar lipids are grouped and coloured based on the charge of their headgroups: neutral, anionic and zwitterionic. Core lipids were extracted together with intact polar lipids and were included in the visualization. The biomass samples were obtained at the end of respective bioreactor batch incubations, solid lines indicate significant relationships (*p* < 0.05) with 0.95 confidence interval in grey.
**Figure S6:** MS^2^ fragmentation spectra of part of the identified archaeols. The spectra of phosphatidylethanolamine‐archaeol (A), phosphatidylethanolamine‐1uns‐hydroxyarchaeol (B), phosphatidylmethylethanolamine‐1uns‐hydroxyarchaeol (C) and phosphatidylmethylethanolamine‐7uns‐hydroxyarchaeol (D) have not been described before and were detected in intact polar lipid extract from the biomass of ‘*Ca*. M. vercellensis’.
**Table S1:** Classification table of intact polar lipids. Intact polar lipids are grouped based on their ability to be charged into neutral, anionic and zwitterionic.
**Table S2:** Table with results of statistical testing.

**Data S1:** Table S1: Classification table of intact polar lipids. Intact polar lipids are grouped based on their ability to be charged into neutral, anionic, and zwitterionic.

**Data S2:** Table S2: Output of linear regression models.

## Data Availability

The data that support the findings of this study are openly available in zenodo at https://zenodo.org/records/19912675, reference number doi:10.5281/zenodo.19912675.
