# Supplementary material for: Long‐Term Acclimation and Membrane Lipid Remodelling Enable Anaerobic Oxidation of Methane at Low‐pH by ‘Ca. Methanoperedens vercellensis’
Source: Environ Microbiol. 2026 Nov 9;28(11):e70460. doi: 10.1111/1462-2920.70460 (PMC13747702; doi:10.1111/1462-2920.70460)
Supplement: Supplementary file 1 [file EMI-28-e70460-s002.docx]

**Supporting Information**

**Long-term acclimation and membrane lipid remodeling enable anaerobic oxidation of methane at low-pH by ‘*Ca*. Methanoperedens vercellensis’**

Vojtěch Tláskal^1 2^, Reinier A. Egas^2^, Wei Wang^2^ ^3^, Xiaoxiao Zhao^4^, Martijn Wissink^2^, Maider J. Echeveste Medrano^2^ ^#^, Kevin W. Becker^5 6^, Felix J. Elling^4 §^*, Cornelia U. Welte^2^*

¹ Institute of Soil Biology and Biogeochemistry, Biology Centre of the Czech Academy of Sciences, Na Sádkách 7, 37005 České Budějovice, Czech Republic.

² Department of Microbiology, Radboud Institute for Biological and Environmental Sciences, Radboud University, Heyendaalseweg 135, 6525AJ Nijmegen, The Netherlands.

³ State Key Laboratory of Urban-rural Water Resource and Environment, School of Environment, Harbin Institute of Technology, Harbin, Heilongjiang Province, China

^4^ Leibniz-Laboratory for Radiometric Dating and Isotope Research, Christian-Albrecht University of Kiel, Ludewig-Meyn-Straße 14, 24118 Kiel, Germany

^5^ GEOMAR Helmholtz Centre for Ocean Research Kiel, Wischhofstr. 1-3, 24148 Kiel, Germany

^6^ Faculty of Biology/Chemistry & MARUM – Center for Marine Environmental Sciences, University of Bremen, Bibliothekstraße 1, 28359 Bremen, Germany

*Corresponding authors: Cornelia U. Welte ([c.welte@science.ru.nl](mailto:c.welte@science.ru.nl)), Felix J. Elling ([felix.elling@geow.uni-heidelberg.de](mailto:felix.elling@geow.uni-heidelberg.de))

**Table of Contents:**

Supplementary Figures 1–6

Supplementary Tables 1–2

**Figures**

**Supplementary Figure 1. The characteristics of the bioreactor with the enrichment culture of ‘*Ca*. M. vercellensis’ during the 225 days of the acidification experiment.** Nitrate added to the bioreactor (in mmol day^-1^, **A**) and concentrations of nitrite (**B**) and nitrate (**C**) measured *in situ* in the bioreactor over the course of the acidification experiment (both in mmol L^-1^).

**Supplementary Figure 2. The decrease of pH of bioreactor medium with ‘*Ca*. M. vercellensis’ enrichment culture.** Individual points represent timepoints at the beginning of bioreactor batch incubations. The numbers denote pH values.

**Supplementary Figure 3. The rate of anaerobic oxidation of methane by the enrichment culture of ‘*Ca*. M. vercellensis’ as a function of time**. The rate of anaerobic oxidation of methane is expressed as the slope of change in ratio of ^45^CO_2_/^44^CO_2_ in the bioreactor headspace per day and it is corrected for dry mass of the bioreactor. Individual bioreactor batch activity assays were performed during the 225 days of the acidification experiment.

**Supplementary Figure 4. The inhibition of ‘*Ca*. M. vercellensis’ by 3-bromopropanesulfonic acid (3-BPS).** ^45^CO_2_/^44^CO_2_ ratio in the headspace of batch incubations with 3-BPS (n = 3 biological replicates) and control bottles (n = 3 biological replicates) was used to assess oxidation of ^13^C-CH₄. Replicates are displayed separately to show the variation of individual batch incubation assays.

**Supplementary Figure 5. The relative fraction of intact polar lipids extracted from the biomass of ‘*Ca*. M. vercellensis’**. Intact polar lipids are grouped and coloured based on the charge of their headgroups: neutral, anionic, and zwitterionic. Core lipids were extracted together with intact polar lipids and were included in the visualisation. The biomass samples were obtained at the end of respective bioreactor batch incubations, solid lines indicate significant relationships (p < 0.05) with 0.95 confidence interval in grey.

**Supplementary Figure 6. MS^2^ fragmentation spectra of part of the identified archaeols.** The spectra of phosphatidylethanolamine-archaeol (**A**), phosphatidylethanolamine-1uns-hydroxyarchaeol (**B**), phosphatidylmethylethanolamine-1uns-hydroxyarchaeol (**C**), and phosphatidylmethylethanolamine-7uns-hydroxyarchaeol (**D**) have not been described before and were detected in intact polar lipid extract from the biomass of ‘*Ca*. M. vercellensis’.

**Tables**

**Supplementary Table 1. Classification table of intact polar lipids.** Intact polar lipids are grouped based on their ability to be charged into neutral, anionic, and zwitterionic.

**Supplementary Table 2. Table with results of statistical testing.**
